# 2-(4-Fluoro­phen­yl)-2-oxoethyl 3-(trifluoro­meth­yl)benzoate

**DOI:** 10.1107/S160053681103947X

**Published:** 2011-10-05

**Authors:** Hoong-Kun Fun, Suhana Arshad, B. Garudachari, A. M. Isloor, Kammasandra N Shivananda

**Affiliations:** a X-ray Crystallography Unit, School of Physics, Universiti Sains Malaysia, 11800 USM, Penang, Malaysia; b Medicinal Chemistry Division, Department of Chemistry, National Institute of Technology-Karnataka, Surathkal, Mangalore 575 025, India; c Schulich Faculty of Chemistry, Technion Israel Institute of Technology, Haifa 32000, Israel

## Abstract

In the title compound, C_16_H_10_F_4_O_3_, the fluoro­form group is disordered over two orientations with an occupancy ratio of 0.834 (4):0.166 (4). The dihedral angle between the two aromatic rings is 20.34 (9)°. In the crystal, C—H⋯O hydrogen bonds link the mol­ecules into layers lying parallel to the *bc* plane.

## Related literature

For background to the chemistry of phenacyl benzoate deriv­atives, see: Huang *et al.* (1996 ▶); Gandhi *et al.* (1995 ▶); Ruzicka *et al.* (2002 ▶); Litera *et al.* (2006 ▶); Sheehan & Umezaw (1973 ▶). For bond-length data, see: Allen *et al.* (1987 ▶). For a related structure, see: Fun *et al.* (2011 ▶).
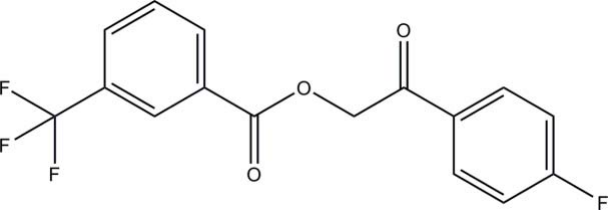


## Experimental

### 

#### Crystal data

C_16_H_10_F_4_O_3_
*M* *_r_* = 326.24
Monoclinic, 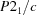

*a* = 14.7694 (19) Å
*b* = 12.1602 (16) Å
*c* = 8.0929 (10) Å
β = 95.886 (2)°
*V* = 1445.8 (3) Å^3^
*Z* = 4
Mo *K*α radiation
μ = 0.14 mm^−1^
*T* = 296 K
0.38 × 0.25 × 0.07 mm

#### Data collection

Bruker SMART APEXII DUO CCD diffractometer
Absorption correction: multi-scan (*SADABS*; Bruker, 2009 ▶) *T*_min_ = 0.950, *T*_max_ = 0.990
18790 measured reflections
4815 independent reflections
2841 reflections with *I* > 2σ(*I*)
*R*_int_ = 0.028

#### Refinement

*R*[*F* ^2^ > 2σ(*F* ^2^)] = 0.057
*wR*(*F* ^2^) = 0.182
*S* = 1.04
4815 reflections
221 parameters
H-atom parameters constrained
Δρ_max_ = 0.34 e Å^−3^
Δρ_min_ = −0.35 e Å^−3^

### 

Data collection: *APEX2* (Bruker, 2009 ▶); cell refinement: *SAINT* (Bruker, 2009 ▶); data reduction: *SAINT*; program(s) used to solve structure: *SHELXTL* (Sheldrick, 2008 ▶); program(s) used to refine structure: *SHELXTL*; molecular graphics: *SHELXTL*; software used to prepare material for publication: *SHELXTL*and *PLATON* (Spek, 2009 ▶).

## Supplementary Material

Crystal structure: contains datablock(s) global, I. DOI: 10.1107/S160053681103947X/hb6418sup1.cif

Structure factors: contains datablock(s) I. DOI: 10.1107/S160053681103947X/hb6418Isup2.hkl

Supplementary material file. DOI: 10.1107/S160053681103947X/hb6418Isup3.cml

Additional supplementary materials:  crystallographic information; 3D view; checkCIF report

## Figures and Tables

**Table 1:** Hydrogen-bond geometry (Å, °)

*D*—H⋯*A*	*D*—H	H⋯*A*	*D*⋯*A*	*D*—H⋯*A*
C5—H5*A*⋯O3^i^	0.93	2.50	3.263 (2)	139
C8—H8*A*⋯O1^ii^	0.97	2.55	3.502 (2)	169

Symmetry codes: (i) 
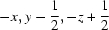
; (ii) 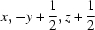.

**Fig. 1.**
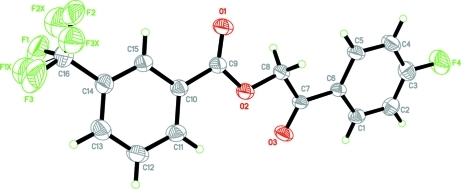
The molecular structure of the title compound, showing 30% probability displacement ellipsoids. Both disordered components are shown.

**Fig. 2.**
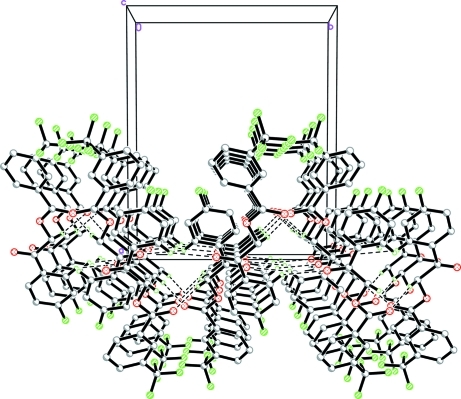
The crystal packing of the title compound. The dashed lines represent the hydrogen bonds. Only the major disordered components are shown.

## supplementary crystallographic information

### Comment

In organic chemistry, phenacyl benzoate is a derivative of an acid formed by reaction between acid and phenacyl bromide. They find applications in the field of synthetic chemistry (Huang *et al.*, 1996; Gandhi *et al.*, 1995) such as synthesis of oxazoles, imidazoles and benzoxazepines. They are also useful for photo-removable protecting groups for carboxylic acids in organic synthesis and biochemistry (Ruzicka *et al.*, 2002; Litera *et al.*, 2006; Sheehan & Umezaw, 1973). Keeping this in view, the title compound was synthesized to study its crystal structure.

In the molecular structure (Fig. 1), the fluoro form group is disordered over two orientations with an occupancy ratio of 0.834 (4):0.166 (4). The two phenyl rings (C1–C6 & C10–C15) make a dihedral angle of 20.34 (9)°. Bond lengths (Allen *et al.*, 1987) and angles are within normal range and are comparable to the related structures (Fun *et al.*, 2011).

The crystal packing is shown in Fig. 2. Intermolecular C5—H5A···O3 and C8—H8A···O1 hydrogen bonds (Table 1) linked the molecules into layers parallel to *bc* plane.

### Experimental

A mixture of 3-(trifluoromethyl)benzoic acid (1.0 g, 0.0052 mol), potassium carbonate (0.78 g, 0.0057 mol) and 2-bromo-1-(4-fluorophenyl)ethanone (1.12 g, 0.0052 mol) in dimethylformamide (10 ml) was stirred at room temperature for 2 h. On cooling, colourless needle-shaped crystals of 2-(4-fluorophenyl)-2-oxoethyl 3-(trifluoromethyl)benzoate begin to separate out. These were collected by filtration and recrystallized from ethanol to yield colourles blocks. Yield: 1.6 g, 93.5%. *M.p*: 369–370 K.

### Refinement

The fluoro form group is disordered over two orientations, with a final refined occupancy ratio of 0.834 (4):0.166 (4). All H atoms were positioned geometrically [C–H = 0.93 or 0.97 Å] and refined using a riding model with *U*_iso_(H) = 1.2 *U*_eq_(C).

### Crystal data

**Table:**

C_16_H_10_F_4_O_3_	*F*(000) = 664
*M**_r_* = 326.24	*D*_x_ = 1.499 Mg m^−^^3^
Monoclinic, *P*2_1_/*c*	Mo *K*α radiation, λ = 0.71073 Å
Hall symbol: -P 2ybc	Cell parameters from 4165 reflections
*a* = 14.7694 (19) Å	θ = 2.2–27.2°
*b* = 12.1602 (16) Å	µ = 0.14 mm^−^^1^
*c* = 8.0929 (10) Å	*T* = 296 K
β = 95.886 (2)°	Block, colourless
*V* = 1445.8 (3) Å^3^	0.38 × 0.25 × 0.07 mm
*Z* = 4	

### Data collection

**Table:**

Bruker SMART APEXII DUO CCD diffractometer	4815 independent reflections
Radiation source: fine-focus sealed tube	2841 reflections with *I* > 2σ(*I*)
graphite	*R*_int_ = 0.028
φ and ω scans	θ_max_ = 31.6°, θ_min_ = 1.4°
Absorption correction: multi-scan (*SADABS*; Bruker, 2009)	*h* = −21→21
*T*_min_ = 0.950, *T*_max_ = 0.990	*k* = −17→17
18790 measured reflections	*l* = −11→11

### Refinement

**Table:**

Refinement on *F*^2^	Primary atom site location: structure-invariant direct methods
Least-squares matrix: full	Secondary atom site location: difference Fourier map
*R*[*F*^2^ > 2σ(*F*^2^)] = 0.057	Hydrogen site location: inferred from neighbouring sites
*wR*(*F*^2^) = 0.182	H-atom parameters constrained
*S* = 1.04	*w* = 1/[σ^2^(*F*_o_^2^) + (0.0745*P*)^2^ + 0.3289*P*] where *P* = (*F*_o_^2^ + 2*F*_c_^2^)/3
4815 reflections	(Δ/σ)_max_ < 0.001
221 parameters	Δρ_max_ = 0.34 e Å^−^^3^
0 restraints	Δρ_min_ = −0.35 e Å^−^^3^

### Special details

**Table:**

Geometry. All e.s.d.'s (except the e.s.d. in the dihedral angle between two l.s. planes) are estimated using the full covariance matrix. The cell e.s.d.'s are taken into account individually in the estimation of e.s.d.'s in distances, angles and torsion angles; correlations between e.s.d.'s in cell parameters are only used when they are defined by crystal symmetry. An approximate (isotropic) treatment of cell e.s.d.'s is used for estimating e.s.d.'s involving l.s. planes.
Refinement. Refinement of *F*^2^ against ALL reflections. The weighted *R*-factor *wR* and goodness of fit *S* are based on *F*^2^, conventional *R*-factors *R* are based on *F*, with *F* set to zero for negative *F*^2^. The threshold expression of *F*^2^ > σ(*F*^2^) is used only for calculating *R*-factors(gt) *etc*. and is not relevant to the choice of reflections for refinement. *R*-factors based on *F*^2^ are statistically about twice as large as those based on *F*, and *R*- factors based on ALL data will be even larger.

### Fractional atomic coordinates and isotropic or equivalent isotropic displacement parameters (Å^2^)

**Table:**

	*x*	*y*	*z*	*U*_iso_*/*U*_eq_	Occ. (<1)
F1	0.41559 (16)	0.37665 (19)	−0.5044 (2)	0.1144 (9)	0.834 (4)
F2	0.44559 (19)	0.25120 (16)	−0.3326 (4)	0.1179 (11)	0.834 (4)
F3	0.53719 (16)	0.3931 (3)	−0.3405 (5)	0.1359 (12)	0.834 (4)
F1X	0.5096 (11)	0.4001 (13)	−0.4146 (18)	0.110 (5)*	0.166 (4)
F2X	0.4251 (10)	0.2913 (17)	−0.413 (2)	0.137 (6)*	0.166 (4)
F3X	0.5187 (7)	0.2898 (8)	−0.2308 (12)	0.096 (3)*	0.166 (4)
F4	−0.27124 (10)	0.35096 (15)	0.5827 (2)	0.1118 (6)	
O1	0.18686 (10)	0.27074 (10)	−0.0004 (2)	0.0756 (4)	
O2	0.15270 (8)	0.43444 (10)	0.10304 (15)	0.0578 (3)	
O3	0.02656 (9)	0.56904 (9)	0.18644 (16)	0.0623 (3)	
C1	−0.11779 (12)	0.51838 (14)	0.3751 (2)	0.0522 (4)	
H1A	−0.1071	0.5922	0.3543	0.063*	
C2	−0.18848 (13)	0.48938 (17)	0.4648 (2)	0.0628 (5)	
H2A	−0.2257	0.5427	0.5050	0.075*	
C3	−0.20243 (14)	0.38007 (19)	0.4931 (3)	0.0707 (5)	
C4	−0.14948 (14)	0.29857 (17)	0.4363 (3)	0.0720 (5)	
H4A	−0.1609	0.2250	0.4577	0.086*	
C5	−0.07894 (13)	0.32825 (14)	0.3467 (2)	0.0577 (4)	
H5A	−0.0423	0.2741	0.3071	0.069*	
C6	−0.06199 (11)	0.43833 (12)	0.31499 (18)	0.0447 (3)	
C7	0.01478 (11)	0.47363 (12)	0.22220 (19)	0.0458 (3)	
C8	0.07752 (12)	0.38408 (13)	0.1730 (2)	0.0523 (4)	
H8A	0.0994	0.3408	0.2696	0.063*	
H8B	0.0451	0.3356	0.0921	0.063*	
C9	0.20286 (12)	0.36718 (13)	0.0189 (2)	0.0520 (4)	
C10	0.27900 (11)	0.42548 (13)	−0.0491 (2)	0.0502 (4)	
C11	0.30026 (13)	0.53430 (15)	−0.0112 (3)	0.0658 (5)	
H11A	0.2676	0.5727	0.0624	0.079*	
C12	0.36985 (16)	0.58564 (18)	−0.0826 (3)	0.0818 (7)	
H12A	0.3833	0.6589	−0.0581	0.098*	
C13	0.41962 (15)	0.52905 (18)	−0.1900 (3)	0.0753 (6)	
H13A	0.4665	0.5639	−0.2379	0.090*	
C14	0.39943 (13)	0.42070 (16)	−0.2259 (2)	0.0604 (4)	
C15	0.32944 (12)	0.36853 (14)	−0.1566 (2)	0.0550 (4)	
H15A	0.3161	0.2953	−0.1820	0.066*	
C16	0.45169 (18)	0.3601 (2)	−0.3439 (4)	0.0866 (7)	

### Atomic displacement parameters (Å^2^)

**Table:**

	*U*^11^	*U*^22^	*U*^33^	*U*^12^	*U*^13^	*U*^23^
F1	0.153 (2)	0.1219 (18)	0.0750 (12)	0.0103 (13)	0.0431 (12)	−0.0150 (11)
F2	0.162 (2)	0.0723 (12)	0.1333 (19)	0.0433 (13)	0.0827 (18)	0.0078 (12)
F3	0.0656 (13)	0.194 (3)	0.156 (3)	−0.0032 (14)	0.0486 (15)	−0.064 (2)
F4	0.0863 (10)	0.1217 (13)	0.1375 (13)	−0.0144 (9)	0.0607 (9)	0.0101 (10)
O1	0.0871 (10)	0.0428 (7)	0.1035 (11)	−0.0067 (6)	0.0413 (8)	−0.0073 (7)
O2	0.0601 (7)	0.0439 (6)	0.0729 (8)	−0.0040 (5)	0.0234 (6)	−0.0052 (5)
O3	0.0732 (8)	0.0360 (6)	0.0797 (8)	−0.0019 (5)	0.0170 (6)	0.0077 (5)
C1	0.0571 (9)	0.0416 (8)	0.0576 (9)	0.0070 (7)	0.0040 (7)	−0.0007 (7)
C2	0.0567 (10)	0.0668 (12)	0.0655 (10)	0.0109 (9)	0.0099 (8)	−0.0044 (9)
C3	0.0556 (11)	0.0800 (14)	0.0791 (13)	−0.0082 (10)	0.0185 (9)	0.0035 (10)
C4	0.0748 (13)	0.0512 (10)	0.0933 (14)	−0.0120 (9)	0.0253 (11)	0.0056 (10)
C5	0.0640 (10)	0.0377 (8)	0.0735 (11)	−0.0022 (7)	0.0171 (8)	−0.0004 (7)
C6	0.0494 (8)	0.0350 (7)	0.0493 (8)	0.0001 (6)	0.0033 (6)	0.0004 (6)
C7	0.0542 (9)	0.0338 (7)	0.0491 (8)	−0.0008 (6)	0.0042 (6)	0.0008 (6)
C8	0.0580 (9)	0.0404 (8)	0.0606 (9)	−0.0023 (7)	0.0164 (7)	0.0015 (7)
C9	0.0572 (9)	0.0425 (8)	0.0574 (9)	0.0022 (7)	0.0117 (7)	0.0004 (7)
C10	0.0503 (9)	0.0429 (8)	0.0581 (9)	0.0028 (6)	0.0090 (7)	0.0009 (7)
C11	0.0630 (11)	0.0483 (9)	0.0894 (13)	−0.0005 (8)	0.0236 (10)	−0.0124 (9)
C12	0.0803 (14)	0.0515 (11)	0.1196 (18)	−0.0159 (10)	0.0387 (13)	−0.0176 (11)
C13	0.0658 (12)	0.0634 (12)	0.1012 (16)	−0.0088 (10)	0.0307 (11)	−0.0018 (11)
C14	0.0562 (10)	0.0586 (10)	0.0685 (11)	0.0035 (8)	0.0172 (8)	−0.0001 (8)
C15	0.0596 (10)	0.0446 (8)	0.0623 (10)	0.0038 (7)	0.0129 (8)	−0.0012 (7)
C16	0.0847 (16)	0.0780 (16)	0.1050 (19)	−0.0056 (13)	0.0472 (14)	−0.0146 (14)

### Geometric parameters (Å, °)

**Table:**

F1—C16	1.368 (4)	C5—C6	1.391 (2)
F2—C16	1.331 (3)	C5—H5A	0.9300
F3—C16	1.323 (3)	C6—C7	1.486 (2)
F1X—C16	1.182 (16)	C7—C8	1.510 (2)
F2X—C16	1.059 (17)	C8—H8A	0.9700
F3X—C16	1.537 (10)	C8—H8B	0.9700
F4—C3	1.355 (2)	C9—C10	1.482 (2)
O1—C9	1.203 (2)	C10—C11	1.387 (2)
O2—C9	1.336 (2)	C10—C15	1.387 (2)
O2—C8	1.4342 (19)	C11—C12	1.379 (3)
O3—C7	1.2126 (18)	C11—H11A	0.9300
C1—C2	1.377 (3)	C12—C13	1.379 (3)
C1—C6	1.395 (2)	C12—H12A	0.9300
C1—H1A	0.9300	C13—C14	1.375 (3)
C2—C3	1.368 (3)	C13—H13A	0.9300
C2—H2A	0.9300	C14—C15	1.380 (2)
C3—C4	1.371 (3)	C14—C16	1.484 (3)
C4—C5	1.377 (3)	C15—H15A	0.9300
C4—H4A	0.9300		
			
C9—O2—C8	115.59 (13)	C12—C11—C10	120.07 (17)
C2—C1—C6	120.80 (16)	C12—C11—H11A	120.0
C2—C1—H1A	119.6	C10—C11—H11A	120.0
C6—C1—H1A	119.6	C13—C12—C11	120.44 (18)
C3—C2—C1	118.20 (17)	C13—C12—H12A	119.8
C3—C2—H2A	120.9	C11—C12—H12A	119.8
C1—C2—H2A	120.9	C14—C13—C12	119.56 (19)
F4—C3—C2	118.5 (2)	C14—C13—H13A	120.2
F4—C3—C4	118.41 (19)	C12—C13—H13A	120.2
C2—C3—C4	123.04 (18)	C13—C14—C15	120.65 (18)
C3—C4—C5	118.40 (18)	C13—C14—C16	119.73 (19)
C3—C4—H4A	120.8	C15—C14—C16	119.59 (19)
C5—C4—H4A	120.8	C14—C15—C10	119.88 (16)
C4—C5—C6	120.66 (17)	C14—C15—H15A	120.1
C4—C5—H5A	119.7	C10—C15—H15A	120.1
C6—C5—H5A	119.7	F2X—C16—F1X	108.5 (12)
C5—C6—C1	118.91 (15)	F2X—C16—F3	123.5 (9)
C5—C6—C7	122.18 (14)	F1X—C16—F2	120.0 (8)
C1—C6—C7	118.90 (14)	F3—C16—F2	111.8 (3)
O3—C7—C6	122.18 (14)	F2X—C16—F1	61.8 (11)
O3—C7—C8	121.35 (14)	F1X—C16—F1	73.2 (7)
C6—C7—C8	116.47 (12)	F3—C16—F1	104.7 (3)
O2—C8—C7	108.49 (12)	F2—C16—F1	100.9 (3)
O2—C8—H8A	110.0	F2X—C16—C14	122.9 (9)
C7—C8—H8A	110.0	F1X—C16—C14	123.9 (8)
O2—C8—H8B	110.0	F3—C16—C14	113.3 (2)
C7—C8—H8B	110.0	F2—C16—C14	114.0 (2)
H8A—C8—H8B	108.4	F1—C16—C14	111.1 (2)
O1—C9—O2	123.44 (16)	F2X—C16—F3X	93.2 (11)
O1—C9—C10	124.35 (16)	F1X—C16—F3X	93.6 (8)
O2—C9—C10	112.20 (14)	F3—C16—F3X	66.5 (4)
C11—C10—C15	119.39 (16)	F2—C16—F3X	56.6 (4)
C11—C10—C9	122.52 (15)	F1—C16—F3X	144.3 (4)
C15—C10—C9	118.08 (15)	C14—C16—F3X	103.9 (4)
			
C6—C1—C2—C3	0.0 (3)	C15—C10—C11—C12	1.2 (3)
C1—C2—C3—F4	179.32 (18)	C9—C10—C11—C12	−177.7 (2)
C1—C2—C3—C4	−0.1 (3)	C10—C11—C12—C13	−0.9 (4)
F4—C3—C4—C5	−179.30 (19)	C11—C12—C13—C14	0.0 (4)
C2—C3—C4—C5	0.1 (3)	C12—C13—C14—C15	0.6 (3)
C3—C4—C5—C6	0.0 (3)	C12—C13—C14—C16	178.9 (2)
C4—C5—C6—C1	−0.1 (3)	C13—C14—C15—C10	−0.3 (3)
C4—C5—C6—C7	178.56 (17)	C16—C14—C15—C10	−178.6 (2)
C2—C1—C6—C5	0.2 (2)	C11—C10—C15—C14	−0.6 (3)
C2—C1—C6—C7	−178.58 (15)	C9—C10—C15—C14	178.38 (16)
C5—C6—C7—O3	175.79 (16)	C13—C14—C16—F2X	−154.8 (13)
C1—C6—C7—O3	−5.5 (2)	C15—C14—C16—F2X	23.5 (14)
C5—C6—C7—C8	−3.7 (2)	C13—C14—C16—F1X	−2.1 (9)
C1—C6—C7—C8	174.96 (14)	C15—C14—C16—F1X	176.3 (9)
C9—O2—C8—C7	−165.10 (14)	C13—C14—C16—F3	32.0 (4)
O3—C7—C8—O2	7.2 (2)	C15—C14—C16—F3	−149.7 (3)
C6—C7—C8—O2	−173.24 (13)	C13—C14—C16—F2	161.3 (3)
C8—O2—C9—O1	0.9 (3)	C15—C14—C16—F2	−20.3 (4)
C8—O2—C9—C10	−179.97 (14)	C13—C14—C16—F1	−85.5 (3)
O1—C9—C10—C11	−173.9 (2)	C15—C14—C16—F1	92.9 (3)
O2—C9—C10—C11	7.0 (2)	C13—C14—C16—F3X	102.0 (5)
O1—C9—C10—C15	7.2 (3)	C15—C14—C16—F3X	−79.6 (5)
O2—C9—C10—C15	−171.99 (15)		

### Hydrogen-bond geometry (Å, °)

**Table:**

*D*—H···*A*	*D*—H	H···*A*	*D*···*A*	*D*—H···*A*
C5—H5A···O3^i^	0.93	2.50	3.263 (2)	139
C8—H8A···O1^ii^	0.97	2.55	3.502 (2)	169

Symmetry codes: (i) −*x*, *y*−1/2, −*z*+1/2; (ii) *x*, −*y*+1/2, *z*+1/2.
